# *Crinum Latifolium* Leave Extracts Suppress Immune Activation Cascades in Peripheral Blood Mononuclear Cells and Proliferation of Prostate Tumor Cells

**DOI:** 10.3797/scipharm.1011-13

**Published:** 2011-04-05

**Authors:** Marcel Jenny, Angela Wondrak, Elissaveta Zvetkova, Nguyen Thi Ngoc Tram, Phan Thi Phi Phi, Harald Schennach, Zoran Culig, Florian Ueberall, Dietmar Fuchs

**Affiliations:** 1 Division of Biological Chemistry, Innsbruck Medical University, Fritz-Pregl-Str. 3, 6020 Innsbruck, Austria; 2 Division of Medical Biochemistry, Innsbruck Medical University, Fritz-Pregl-Str. 3, 6020 Innsbruck, Austria; 3 Institute of Experimental Pathology and Parasitology, Bulgarian Academy of Sciences, 1113 Sofia, Bulgaria; 4 Thien Duoc Company Ltd., 51/4/9 Thanh Thai St. Ward 14, District 10, Ho Chi Minh City, Vietnam; 5 Hanoi Medical University, Ton That Tung Street 1, Hanoi, Vietnam; 6 Central Institute of Blood Transfusion and Immunology, University Hospital Innsbruck, Anichstraße 35, 6020 Innsbruck, Austria; 7 Department of Urology, Innsbruck Medical University, Anichstraße 35, 6020 Innsbruck, Austria

**Keywords:** *Crinum latifolium* (L.), Peripheral blood mononuclear cells, Indoleamine 2, 3-dioxygenase, Prostate cells, Proliferation

## Abstract

Plants of the genus *Crinum* (*Amaryllidaceae*) are widely used in folk medicine in different tropical and subtropical regions around the world. The Indian species *Crinum latifolium* (L.) was traditionally used to treat rheumatism, fistula, tumors, earaches, rubefacient, tubercle and whitlow. In Vietnamese and Chinese traditional medicine *Crinum latifolium* preparations are used until nowadays because of their antiviral and antitumor properties. In this study, we demonstrate potent *in vitro* antioxidant activity of an aqueous *Crinum latifolium* extract *by an* oxygen radical absorbance capacity (ORAC) value of 1610 ± 150 μmol Trolox equivalents/g. Furthermore, significant anti-inflammatory effects of this extract were shown by its potential to suppress indoleamine 2,3-dioxygenase (IDO) mediated tryptophan degradation in unstimulated- and mitogen-stimulated PBMC at IC_50_ doses of 241 ± 57 μg/ml and 92 ± 20 μg/ml, respectively. Concentrations of the immune activation marker neopterin were slightly diminished in unstimulated PBMC, whereas a dose-dependent inhibition of neopterin formation was observed in mitogen-stimulated PBMC (IC_50_ = 453 ± 86 μg/ml). Additionally, we measured also dose-dependent inhibitory effects of this aqueous *Crinum latifolium* extract on cell proliferation of highly metastatic human prostate carcinoma PC3 cells (IC_50_ = 4.5 ± 0.8 mg/ml), androgen-sensitive prostate adenocarcinoma LNCaP cells (IC_50_ =2.3 ± 0.1 mg/ml), and benign prostate hyperplasia BPH-1 cells (IC_50_ = 2.1 ± 0.04 mg/ml). We conclude that both effects, inhibition of tumor cell growth and recovery of immune functions, are important for the antitumor properties of *Crinum latifolium*.

## Introduction

Plants of the genus *Crinum* (*Amaryllidaceae*) comprise about 130 species which are pantropically distributed and widely used in folk medicine in different geographical regions around the world. Extracts of *Crinum* species are reported to exert antitumor, immunostimulating, analgesic, antiviral, antibacterial and antifungal effects, which have been mainly attributed to the more than 150 alkaloids of the *Amaryllidaceae* type present in *Crinum* species [1, 2]. The Indian species *Crinum latifolium* (L.) was traditionally used to treat rheumatism, fistula, tumors, earaches, rubefacient, tubercle and whitlow [3, 4]. In Vietnamese and Chinese traditional medicine hot aqueous extracts of *Crinum latifolium* are used until nowadays because of its antiviral and antitumor activity, especially to treat prostate cancer [5, 6]. Scientific evidence for an antitumor effect of *Crinum latifolium* constituents emerged from an aqueous extract of *Crinum latifolium* leaves, which was found to retard growth of chemically induced 20-methylcholanthrene tumors in rats [7]. The same extract was also shown to activate and stimulate T-cell proliferation of human peripheral blood mononuclear cells (PBMC) and also *in vivo* following treatment of Balb/c mice [8]. Recent *in vitro* studies in our laboratory showed that hot and cold aqueous extracts of *Crinum latifolium* were highly active to suppress mitogen- and interferon (IFN)-γ-induced formation of the pteridine derivative neopterin in PBMC [9].

During the Th-1 type immune response, activated cells release large amounts of cytokines such as interleukin-(IL)-2 or IFN-γ. Pro-inflammatory IFN-γ is an important mediator of the innate and adaptive immune response triggering a variety of physiological and cellular responses e.g. induction of high amounts of antimicrobial and cytocidal reactive oxygen species (ROS) by macrophages and other cells [10]. In human macrophages, IFN-γ induces also the enzyme indoleamine 2,3-dioxygenase (IDO), which catalyses the rate-limiting step of tryptophan degradation via the kynurenine pathway, and formation of the immune activation marker neopterin, via induction of the enzyme guanosine-triphosphate-(GTP)-cyclohydrolase [11, 12]. IDO plays a central role in the suppression of intracellular bacteria and viruses during an antimicrobial immune response and also growth of malignant cells, as ongoing tryptophan degradation limits protein biosynthesis due to deprivation of this essential amino acid [13, 14]. More recently, it has been demonstrated *in vitro* that also T cell proliferation is inhibited efficiently by IDO [15, 16]. Increased tryptophan degradation and neopterin production develop in patients during diseases which are associated with Th1-type immune activation such as infections, autoimmune diseases, malignant disorders, and during allograft rejection episodes [11, 17, 18]. In patients, accelerated tryptophan degradation was found to parallel, and even to predict, the future course of several clinical conditions, including HIV infection, malignancy and autoimmune syndromes such as rheumatoid arthritis [18–20].

In this study, we evaluated the effects of powdered *Crinum latifolium* leaves extracted in water on the T-cell/macrophage interplay by its influence on PBMC stimulated with phytohaemagglutinin (PHA), which activates formation of IFN-γ in T-cells and subsequently tryptophan degradation and neopterin production in macrophages [21]. In an approach to measure the antioxidant capacity of this *Crinum latifolium* extract, we applied the oxygen radical absorption capacity (ORAC) assay. In addition, antiproliferative effects of this *Crinum latifolium* extract were analyzed in human benign prostate hyperplasia BPH-1 cells, highly metastatic prostate carcinoma PC3 cells, and androgen-sensitive prostate adenocarcinoma LNCaP cells.

## Results and Discussion

### Antioxidant activity in vitro

The antioxidant capacity of *Crinum latifolium* extracted in water was evaluated using the ORAC assay, which measures the direct capacity of chain-breaking antioxidants based on the hydrogen atom transfer mechanism. *Crinum latifolium* showed potent peroxyl-radical scavenging capacity *in vitro*, with an ORAC value of 1610 ± 150 μmol TE/g. This ORAC value appears to be remarkably high in comparison to the ORAC values of other herbs. In a study of Liao et al. e.g., analysis of water extracts of 45 herbs traditionally used in chinese medicine revealed a very broad range of activity in the ORAC assay (40–1990 μmol TE/g herbs), of which only two herbal extracts (*Spatholobus suberectus*: 1990 μmol TE/g; *Sanguisorba officinalis*: 1940 mmol TE/g) showed a higher ORAC value than the *Crinum latifolium* extract analyzed in this study [22].

### Anti-inflammatory activity

The aqueous extract of *Crinum latifolium* produced a strong anti-inflammatory effect on mitogen stimulated PBMC by counteracting both, mitogen-induced activation of IDO enzyme activity and secretion of neopterin, which directs to a down-regulatory effect of *Crinum latifolium* constituents on activated T-cells and production of IFN-γ In resting PBMC, the *Crinum latifolium* extract also suppressed tryptophan degradation mediated by spontaneous IDO activity and slightly diminished neopterin production. Suppression of IFN-γ formation and associated down-stream pathways agrees well with earlier findings by us and others, that compounds and extracts with antioxidant capacities (such as vitamin C and E, resveratrol, wine or extracts of cacao, green or black tea) exhibit strong suppressive effects on stimulated PBMC, similarly as the *Crinum latifolium* extract used in this study [9, 23–27].

The supernatants of unstimulated PBMC, cultivated for 48 hours under standard cultivation conditions, contained 21.1 ± 1.6 μmol/L tryptophan and 2.4 ± 0.6 μmol/L kynurenine resulting in a kynurenine to tryptophan ratio (kyn/trp) of 119 ± 15 μmol/mmol, as a measure of spontaneous IDO activity (Table 1). Upon treatment of PBMC with PHA [10 μg/ml] for 48 hours, tryptophan content decreased to 2.6 ± 1.0 μmol/L whereas kynurenine concentrations increased concomitantly to 9.9 ± 0.7 μmol/L, indicating an approximately 40-fold increase of IDO activity (4658 ± 832 μmol/mmol; Table 1). At the same time, neopterin concentrations raised from 4.7 ± 0.5 nmol/L in unstimulated cultures to 16.2 ± 1.8 nmol/L in PHA-stimulated PBMC cultures. Viability of cells was not affected by the treatment with 10 μg/ml PHA for 48 hours (data not shown).

**Fig. 1.**
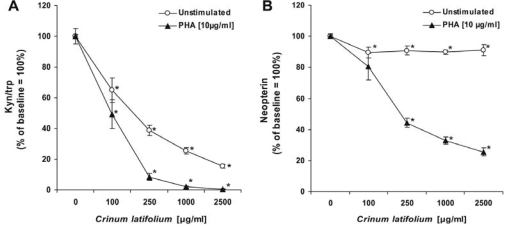
A) Kynurenine to tryptophan ratio (Kyn/trp) and B) neopterin concentrations expressed as % of baseline in the supernatants of unstimulated (open circles) and phytohaemagglutinin (PHA)-stimulated PBMC (closed triangles) pretreated or not with increasing concentrations of *Crinum latifolium* leave extract for 48 hours. Results shown are the mean values ± S.E.M. of four independent experiments run in duplicates (*p <0.05).

Treatment of PBMC with increasing concentrations of *Crinum latifolium* extracts for 48 hours, suppressed tryptophan degradation mediated by IDO activity (kyn/trp) in a dose-dependent manner in unstimulated- and PHA-stimulated cultures with IC_50_'s of 241 ± 57 μg/ml (∼1.2 μg/ml total alkaloids; 0.07 μg/ml crinamidine) and 92 ± 20 μg/ml (∼0.5 μg/ml total alkaloids; 0.03 μg/ml crinamidine), respectively (Fig. 1A). At doses of 250 μg/ml, the extract almost completely suppressed mitogen-induced IDO activity (kyn/trp; Fig. 1A). Measurement of neopterin concentrations showed, that the extract only slightly, but significantly reduced neopterin production in unstimulated PBMC supernatants, whereas PHA-stimulated neopterin formation was suppressed in a dose-dependent manner with an IC_50_ of 453 ± 86 μg/ml (∼2.3 μg/ml total alkaloids; 0.13 μg/ml crinamidine; Fig. 1B). No influence on the viability of PBMC, even at the highest concentrations tested, could be detected after 48 hours of treatment (data not shown).

### Antiproliferative activity

**Fig. 2.**
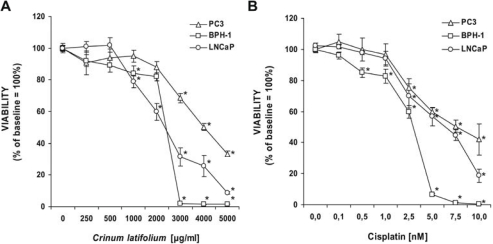
Viability of PC3- (open triangles), BPH-1- (open squares) and LNCaP-cells (open circles) after treatment with or without increasing concentrations of *Crinum latifolium* leave extract (A) or cisplatin (B) for 72 hours, expressed as % of baseline. Results shown are the mean values ± S.E.M. of three independent experiments run in duplicates (*p <0.05).

Treatment of human prostate carcinoma PC3 and LNCaP cells, and also benign prostate hyperplasia BPH-1 cells with water extracts of *Crinum latifolium*, showed a significant and dose-dependent inhibition of cell growth after a culture period of 72 hours (Fig. 2A). Parallel measurements of growth inhibition after treatment of cells with cisplatin were used as a positive control (Figure 2B). The most sensitive cell line affected by these treatments were BPH-1 cells, whose proliferation was inhibited with an IC_50_ of 2120 ± 41 μg/ml (∼10.6 μg/ml total alkaloids; 0.6 μg/ml crinamidine) after treatment with *Crinum latifolium* extract and with an IC_50_ of 2.3 ± 0.3 nM after treatment with cisplatin.

The growth of PC3 and LNCaP cells was suppressed by the *Crinum latifolium* extract at IC_50_-doses of 4540 ± 791 μg/ml (∼22.7 μg/ml total alkaloids; 1.3 μg/ml crinamidine) and 2344 ± 148 μg/ml (∼11.7 μg/ml total alkaloids; 0.7 μg/ml crinamidine), respectively. Cisplatin showed potent antiproliferative effects on PC3 and LNCaP cells at IC_50_-doses of 6.3 ± 0.8 nM and 5.5 ± 0.9 nM, respectively.

It is unclear, which compounds of the *Crinum latifolium* leave extract are responsible for the effects on stimulated PBMC or proliferation of prostate cells. Obviously, the active compounds present in our leave extract of *Crinum latifolium*, were well soluble in water and it appears that antioxidant compounds (represented by a high ORAC value of the extract) and/or specific alkaloids could play a more relevant role. Phytochemical analysis of *Crinum* species revealed more than 170 different compounds, most of which are alkaloids of the *Amaryllidaceae* type, which exhibit a wide range of biological activity such as analgesic, central nervous system, antitumor, and antiviral effects [1, 28]. Although chemical analysis of *Crinum latifolium* alkaloids have been performed mainly on bulbs, which contain the highest concentrations of alkaloids, the presence of alkaloids, especially of the crinane type e.g. crinamine, crinamidine, crinafoline and crinafolidine, were also detected in water extracts of *Crinum latifolium* leaves [29]. The extract used in this study was standardized to contain 0.3 μg crinamidine/mg of dry extract. With respect to the results obtained in this study, we speculate on a possible involvement of such crinane-type **Amaryllidaceae** alkaloids, since they have been shown earlier to possess anticancer activity by inducing apoptosis and at the same time exhibit low toxicity to healthy cells, such as mono-nucleated blood cells [30, 31].

Th1-type cytokine IFN-γ induces activation of IDO in macrophages as well as in other cells including tumor cells such as prostate, colorectal, pancreatic and cervical carcinomas, some of which have been shown to constitutively express IDO [32]. Although IFN-γ production and activation of IDO aims to inhibit the growth of malignant cells and should primarily contribute to tumor rejection, IDO also attenuates T-cell proliferation and consequently the immune response. Thus, beside its role in antimicrobial host defense, IDO plays also an important role as an effector mechanism of the antitumor immune response [33, 34]. However, this immunosuppressive aspect of IDO may enable tumor cells to evade and overcome control by the immune system. Thereby, IDO activity could contribute to the development of immunodeficiency – a major reason for disease progression and death, especially in the latter course of cancer.

A beneficial effect of IDO suppression on tumor growth by specific inhibitors was shown in various animal models. Friberg et al. demonstrated an enhancing effect of the small molecule inhibitor 1-methyl tryptophan (1MT) on T-cell activity *in vitro* and additionally, a capacity of 1MT to retard growth of Lewis lung carcinoma (LLC) cells in syngeneic mice [35]. Uyttenhove et al. showed that ectopic expression of IDO by immunogenic mouse tumor cells promotes tumor growth in preimmunized mice, which could partially be reversed by systemic treatment with 1MT [33]. Using the MMTV-neu/HER2 transgenic mouse model of breast cancer, Muller and coworkers showed also that 1MT slightly retarded tumor outgrowth, whereas combinatory treatment with 1MT and paclitaxel or other chemotherapeutics caused rapid regression of established tumors, which was abrogated in athymic or T-cell depleted mice [36]. Further IDO inhibitors such as methylthiohydantoin-tryptophan (MTH-trp) or the phytoalexin brassinin were also shown to exert a similar pattern of antitumor activity [36–38].

In conclusion, the finding of a strong suppressive effect of *Crinum latifolium* leave extract on IDO activity in stimulated and resting PBMC, may represent a new aspect in the understanding of the antitumor activities of *Crinum latifolium*. In addition, an inhibitory effect on prostate tumor cells was demonstrated. In the aggregate, both effects, inhibition of tumor cell growth and recovery of immune functions, may contribute to the reported beneficial effects of *Crinum latifolium* in the treatment of prostate tumors. Further studies are needed to demonstrate whether our *in vitro* findings can be transferred to the *in vivo* situation in patients.

## Experimental

### Preparation of the aqueous Crinum latifolium extract

**Fig. 3.**
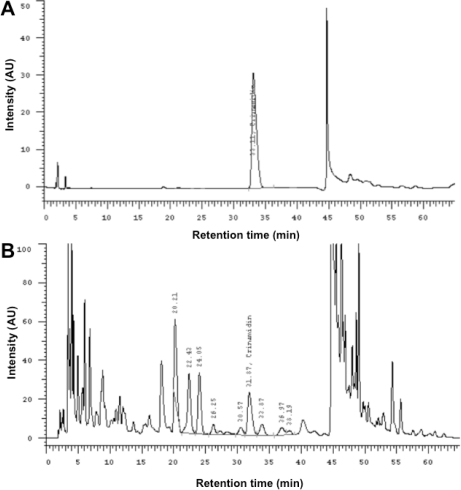
HPLC chromatogram of crinamidine standard (RT 33 min; A), and the dry extract of *Crinum latifolium* leaves (B).

The dry extract of *Crinum latifolium* leaves from CriLa® capsules (250 mg), obtained by the Thien Duoc Company Ltd. (Ho Chi Minh City, Vietnam), was standardized with reference to the amount of crinamidine content (70 μg/capsule) using reverse phase high performance liquid chromatography (HPLC; Figure 3). Content of total alkaloids was also measured by HPLC and calculated with reference to lycorin, which revealed 1.25 ± 0,13 mg total alkaloids/capsule. Leaves from *Crinum latifolium* L. were collected in April and July at the District of Go Vap, Ho Chi Min City, Vietnam. A voucher specimen is kept at the Nguyen Thi Ngoc Tkam Herbarium. The identity of the plant was confirmed by Prof. Dr. Sc. Tkan Cong Khanh, Department of Botany, Hanoi College of Pharmacy, Center for Research and Development of Ethnomedicinal Plants (CREDEP). In this study, 250 mg of the dry powder of a CriLa® capsule was extracted for 1 hour using 50 ml of cell culture media (RPMI) at room temperature. Thereafter, the extract was sterile filtered and stored at room temperature until analysis.

### Cell culture

PBMC were isolated from whole blood obtained from healthy donors, of whom informed consent was obtained that their donated blood unit was used for scientific purposes if not otherwise used. Separation of blood cells was performed using density centrifugation (Lymphoprep, Nycomed Pharma AS, Oslo, Norway). After isolation, PBMC were washed three times in phosphate buffered saline containing 0.2% EDTA [0.5 mmol/L]. Cells were maintained in RPMI 1640 supplemented with 10% heat-inactivated fetal calf serum (Biochrom, Berlin, Germany), 1% of 200 mmol/L glutamine (Serva, Heidelberg, Germany) and 0.1% of gentamicin (50 mg/ml, Bio-Whittaker, Walkersville, MD) in a humidified atmosphere containing 5% CO_2_ for 48h. This procedure was observed earlier to reveal best reproducible results when applied for testing of anti-inflammatory effects of compounds or drugs [39]. Average tryptophan content in the supplemented RPMI 1640 medium was 31.5 μmol/L. For each of the four experiments run in duplicates, PBMC were freshly prepared. Isolated PBMC were plated at a density of 1.5 ×10^6^ cells/ml in supplemented RPMI 1640, preincubated for 30 minutes with or without aqueous *Crinum latifolium* leave extracts and stimulated or not with 10 μg/ml PHA for 48h. Prostate carcinoma PC-3 cells were grown in Coon's F12 Modified Medium (Biochrom, Berlin, Germany) supplemented with 7% FCS, 4 mM L-glutamine and 50 μg/ml gentamycin. Benign prostatic hyperplasia (BPH-1)- and androgen-sensitive human prostate adenocarcinoma LNCaP cells were grown in RPMI 1640 (Biochrom, Berlin, Germany) supplemented with 10% FCS, 4 mM L-glutamine and 50 μg/ml gentamycin. Cells were maintained in a humidified atmosphere containing 5% CO_2._

### Oxygen radical absorbance capacity (ORAC) assay

The capacity of the aqueous *Crinum latifolium* extract to scavenge peroxyl radicals was evaluated by the ORAC assay using the protocol of Ou. et al. [40]. Briefly, the final assay mixture (0.2 ml total volume) contained 6.3 × ^−8^ M fluorescein (AnaSpec, USA) as a target of free radical attack and 1.9 × 10^−2^ M 2,2′-azobis(2-amidinopropane) dihydrochloride (AAPH, Wako Chemicals, Germany), which decomposes in aqueous solutions at 37°C and generates alkyl radicals (R′•) that are converted into the corresponding peroxyl radicals (R′OO•) in the presence of oxygen. 75 mM phosphate buffer served as a blank, and 6-hydroxy-2,5,7,8-tetramethylchroman-2-carboxylic acid (Trolox, a synthetic water-soluble analogue of vitamin E, Sigma-Aldrich, Austria) was used as the control standard. The fluorescence of fluorescein was recorded at 37°C by a fluorometer (Fluoroscan Ascent, Labsystems; 485/535nm). During an assay period of 35 min the decrease of fluorescence was measured in minute intervals after the addition of AAPH. Final results were calculated using the differences between areas under the fluorescein decay curves (AUC) of blank and sample. The protective effect of a putative antioxidant was calculated by comparing the AUC of the sample with that of Trolox. The final ORAC values were expressed as μmol Trolox equivalents per gram of dried powder (μmol TE/g).

### Measurement of tryptophan, kynurenine and neopterin concentrations

After incubation of cells for 48h, supernatants were harvested by centrifugation and tryptophan and kynurenine concentrations were determined by HPLC using 3-nitro-L-tyrosine as an internal standard [41]. To estimate IDO enzyme activity, kyn/trp was calculated and expressed as μmol kynurenine/mmol tryptophan. Neopterin concentrations were determined by an enzyme-linked immunosorbent assay (ELISA; BRAHMS, Hennigsdorf/Berlin, Germany) according to the manufacturer’s instructions with a detection limit of 2 nmol/L.

### Measurement of cell viability

After incubation of PBMC with increasing concentrations of *Crinum latifolium* extract, the cells were either left untreated or stimulated with PHA [10 μg/ml] for 48 hours. Cell viability was measured by reduction of the tetrazole 3-(4,5-dimethylthiazol-2-yl)-2,5-diphenyltetrazolium bromide (MTT) (Sigma Aldrich, Vienna, Austria), which is reduced to purple formazan in living cells, and by trypan blue exclusion method in three experiments done in triplicate. No toxicity of the *Crinum latifolium* extract could be observed in PBMC at the concentration range applied [0.1–2.5 mg/ml]. After incubation of PC3-, BPH-1- or LNCaP-cells with increasing concentrations of *Crinum latifolium* extract [0.25–5 mg/ml] or cisplatin [0.1–10 nM] for 72 hours, cell viability was measured with Cell Titer Blue reagent (Promega, Mannheim, Germany), which measures metabolic activity of living cells by reduction of resazurin to resofurin. These experiments were performed in three independent experiments done in duplicates.

### Statistical analysis

For statistical analysis, the Statistical Package for the Social Sciences (version 14 SPSS, Chicago, Ill, USA) was used. Because not all data sets showed normal distribution, for comparison of grouped data non-parametric Friedman test and Wilcoxon signed ranks test were applied. P-values below 0.05 were considered to indicate significant differences.

## Figures and Tables

**Tab. 1.** Concentrations of tryptophan, kynurenine, neopterin and kyn/trp in the supernatant of unstimulated and phytohaemagglutinin (PHA; 10 μg/ml)-stimulated PBMC.

	**Unstimulated^a^**	**PHA [10 μg/ml]^a^**
Tryptophan [μmol/L	21.2 ± 1.6	2.6 ± 1.0**
Kynurenine [μmol/L]	2.4 ± 0.6	9.9 ± 0.7**
Kyn/trp [μmol/mmol]	119 ± 15	4658 ± 832**
Neopterin [nmol/L]	4.7 ± 0.5	16.2 ± 1.8**

a mean of 4 runs ± S.E.M.

** p <0.005, compared to unstimulated cells.
